# Experimental Determination of Material Behavior Under Compression of a Carbon-Reinforced Epoxy Composite Boat Damaged by Slamming-like Impact

**DOI:** 10.3390/polym18020173

**Published:** 2026-01-08

**Authors:** Erkin Altunsaray, Mustafa Biçer, Haşim Fırat Karasu, Gökdeniz Neşer

**Affiliations:** 1 Institute of Marine Sciences and Technology, Dokuz Eylül University, 35330 Izmir, Türkiye; erkin.altunsaray@deu.edu.tr; 2 Department of Industrial Design, Faculty of Art and Design, Yaşar University, 35100 Izmir, Türkiye; mustafa.bicer@yasar.edu.tr; 3 Mechanical Engineering Department, Engineering Faculty, Dokuz Eylül University, 35390 Izmir, Türkiye; firat.karasu@deu.edu.tr

**Keywords:** carbon-reinforced epoxy laminated composite (CREC), compression after impact (CAI) test, high-speed marine vehicles’ structural design, aging in marine environment

## Abstract

Carbon-reinforced epoxy laminated composite (CREC) structures are increasingly utilized in high-speed marine vehicles (HSMVs) due to their high specific strength and stiffness; however, they are frequently subjected to impact loads like slamming and aggressive environmental agents during operation. This study experimentally investigates the Compression After Impact (CAI) behavior of CREC plates with varying lamination sequences under both atmospheric and accelerated aging conditions. The samples were produced using the vacuum-assisted resin infusion method with three specific orientation types: quasi-isotropic, cross-ply, and angle-ply. To simulate the marine environment, specimens were subjected to accelerated aging in a salt fog and cyclic corrosion cabin for periods of 2, 4, and 6 weeks. Before and following the aging process, low-velocity impact tests were conducted at an energy level of 30 J, after which the residual compressive strength was measured by CAI tests. At the end of the aging process, after the sixth week, the performance of plates with different layer configuration characteristics can be summarized as follows: Plates 1 and 2, which are quasi-isotropic, exhibit opposite behavior. Plate 1, with an initial toughness of 23,000 mJ, increases its performance to 27,000 mJ as it ages, while these values are around 27,000 and 17,000 mJ, respectively, for Plate 2. It is thought that the difference in configurations creates this difference, and the presence of the 0° layer under the effect of compression load at the beginning and end of the configuration has a performance-enhancing effect. In Plates 3 and 4, which have a cross-ply configuration, almost the same performance is observed; the performance, which is initially 13,000 mJ, increases to around 23,000 mJ with the effect of aging. Among the options, angle-ply Plates 5 and 6 demonstrate the highest performance with values around 35,000 mJ, along with an undefined aging effect. Scanning Electron Microscopy (SEM) and Energy-Dispersive X-ray Spectroscopy (EDS) analyses confirmed the presence of matrix cracking, fiber breakage, and salt accumulation (Na and Ca compounds) on the aged surfaces. The study concludes that the impact of environmental aging on CRECs is not uniformly negative; while it degrades certain configurations, it can enhance the toughness and energy absorption of brittle, cross-ply structures through matrix plasticization.

## 1. Introduction

Because of their high specific strength (strength-to-weight ratio), stiffness, and superior resistance to environmental agents at sea (seawater, UV, rapid temperature changes, marine organisms), carbon-reinforced epoxy laminated composite (CREC) structures are the fastest-growing type of fiber-reinforced polymer (FRP) composites in the marine industry. Carbon-reinforced epoxy composites (CFRP) are distinguished by their exceptional specific stiffness and strength; however, they are often compared with glass fiber (GFRP) and basalt fiber (BFRP) reinforced polymers to balance performance and cost. While GFRP is the industry standard for cost-effective applications, BFRP has emerged as an eco-friendly alternative offering superior chemical resistance and higher thermal stability than glass. The choice of matrix also plays a pivotal role; thermoset resins, like the epoxy used in this study, provide excellent dimensional stability and fiber adhesion, though they are inherently brittle and non-recyclable. In contrast, thermoplastic matrices offer enhanced fracture toughness and recyclability but often require higher processing temperatures and face challenges in fiber wetting compared to their thermoset counterparts. In marine environments, the superior fatigue life of carbon fibers combined with the high chemical resistance of epoxy systems typically outperforms glass or basalt-based alternatives, despite the increasing interest in the impact resistance of thermoplastic-based hybrids [1,2,3].

CREC is primarily used in the construction of the hull and internal structural elements of high-speed marine vehicles (HSMV). HSMVs are mainly used for military, recreational, or ambulance services. These vehicles must withstand high impact loads like slamming and grounding during operation. These loads cause damage in the location they affect or, more seriously, damage that will generally disrupt the structural integrity. Even when damaged, HSMVs must remain functional enough to reach a safe coastal structure. For this, the dynamic response of composites to the impacts should be figured out to improve the reliability of HSMVs’ structural elements.

Similarly to other materials, impacts experienced by laminate composites can be divided into three: (1) High velocity (50–1000 m/s): causes expanding pressure waves; (2) Medium velocity (10–50 m/s): causes bending and shear waves; and (3) Low velocity (<10 m/s): considered quasi-static impact [4].

A primary factor that characterizes a slamming event is the entry velocity with which the hull penetrates the surface. Theoretical studies show that slamming pressure increases with the square of the entry velocity [5,6]. Experimental studies by Camilleri, Taunton, and Temareli on GRP hull sections and by Rosen and Garme on HSMVs support this approach [7,8].

Studies show vertical slamming speeds are generally 3–7 m/s, reaching a maximum of 10 m/s. The vertical speeds of ships in grounding are also of similar sizes [9]. In this case, the impacts caused by ship movements in the design of ship structures fall into the low-speed, quasi-static impact class.

Composite impact damage is complex. Controlled experiments are the most effective way to simplify and study this phenomenon. Test evaluation is based on damage morphology, sample characteristics, and support conditions [10,11]. As is known, damages under impact load in FRP composites appear as matrix cracking, fiber breakage, and/or delamination. As the impact energy increases, penetration and excessive local shear damage are also observed.

Low-velocity impact damage is not easily detectable, and the overall weakness it creates in the structure will be noticed quite late. CAI tests (ASTM D7137) [12] are room-temperature uniaxial compression tests. They are widely used to measure the residual strength of damaged components. For the impact and CAI performances of FRPs, theoretical approaches and models have been developed to predict interlaminar and intralaminar damages based on large-scale experimental studies.

Marine composites are exposed to complex environmental agents. These effects reduce the hull’s long-term load capacity and safety. Environmental aging is magnified in hydrophilic polymer-based composites [13]. Water molecules travel through micro-voids, cracks, or fiber/matrix interfaces [14].

Impact resistance degradation under hydrothermal aging is emphasized, though rare in the literature. Studies on this subject have reached three different conclusions:

(1) Aging reduces impact resistance by creating internal defects. This was found through thermal aging, TAI tests, and burst tests [15,16,17,18,19].
(2) Hydrothermal aging has no significant effect on impact resistance. This was shown by ultrasonic analysis, water immersion, and CAI tests [20,21,22].
(3) Hydrothermal aging has a positive effect on impact resistance.

The polymer matrix is the component most affected by environmental wear. Moisture causes chemical degradation at the fiber/matrix interface, weakening the bond [22,23,24].

It is also indicated that the mechanical behavior of CFRP in hydrothermal environments is primarily governed by the competing mechanisms of matrix plasticization and interfacial degradation. Water ingress typically leads to matrix swelling and a reduction in the glass transition temperature (T_g_), a phenomenon known as plasticization, which softens the matrix and can paradoxically increase impact toughness by enhancing ductility. However, prolonged exposure and salt accumulation generally deteriorate the fiber-matrix interfacial bonding and induce micro-cracking due to osmotic pressure, resulting in a significant decline in stiffness and residual compressive strength (CAI). Recent studies confirm that while some toughening may occur initially, the irreversible damage to the interphase region ultimately compromises the structural integrity of marine composites under compressive loads. Matrix plasticization and interface failures reduce mechanical performance and strength [25].

This study performed CAI tests on various plates under atmospheric and marine conditions. The combined effect of composite sequence design and environmental conditions on CAI performance was discussed comparatively using Scanning Electron Microscopy (SEM) and Energy-Dispersive X-ray Spectroscopy (EDS) analysis. In summary, this study addresses a critical gap in the literature regarding the performance of CREC in marine environments, where they are simultaneously exposed to impact loads and aggressive environmental aging. Although the degradation of these materials under hydrothermal conditions is a known concern for HSMVs, focused research on how such aging specifically affects impact resistance remains rare. Furthermore, existing studies present contradictory conclusions, variously suggesting that aging can reduce, have no effect on, or even improve impact resistance. This research fills these voids by experimentally investigating the CAI behavior of CREC plates with various lamination sequences—quasi-isotropic, cross-ply, and angle-ply—under controlled accelerated aging conditions. By examining the combined influence of stacking sequences and environmental wear, the study provides essential structural design data for developing reliable composite marine vessels that can withstand severe operational conditions.

## 2. Experimental Section

### 2.1. Preparation of the Samples

**Figure 1:**
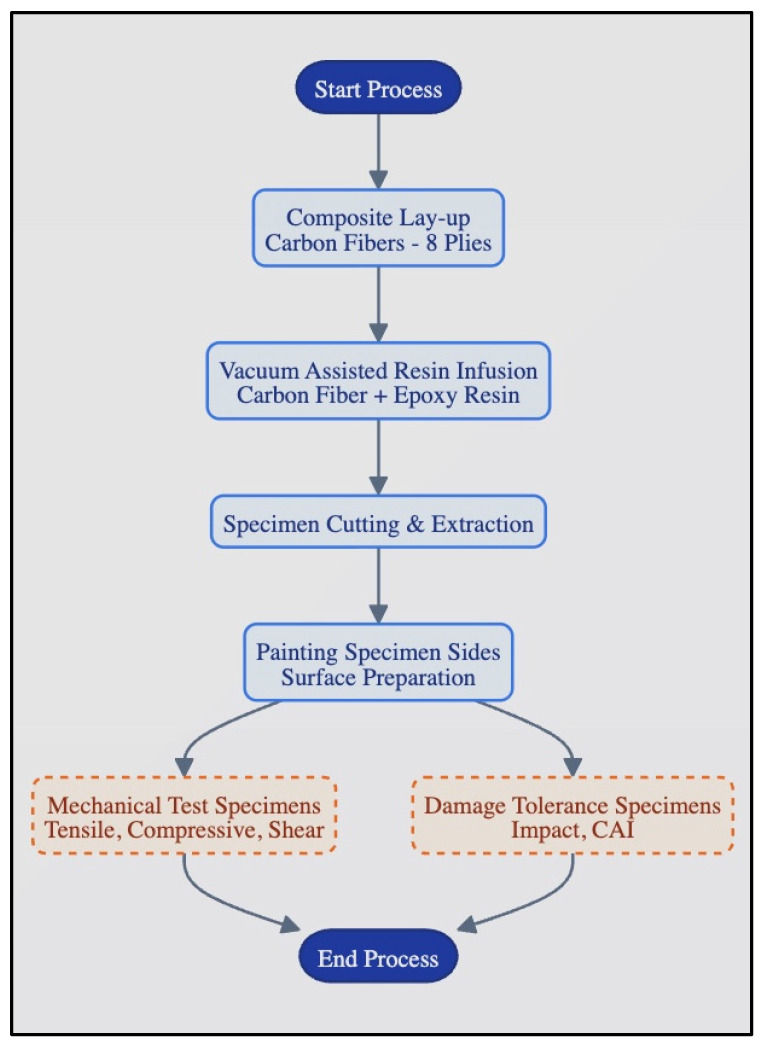
Test processes in the study (Continuous boxes indicated the preparation of the specimens, and dashed boxes indicated tests).

To form composite plates, DowAksa 24K-A42-1600 Tex carbon fibers (Yalova, Turkey) were used as reinforcement, and epoxy resin coded FBRMAKRES-11564 (Yalova, Turkey) with a hardener coded FBRMAKHARD-13487 (Yalova, Turkey) were also used in a special vacuum table with temperature and time adjustment (for 30 min at 50 °C and 8 h at 80 °C) in the vacuum-assisted resin infusion method in the Dokuz Eylul University Mechanical Engineering Composite Materials Lab. The physical and mechanical properties of elements of composite plates given in the manufacturer’s catalog [26] are shown in Table 1. The testing processes applied in this study are summarized in the diagram in Figure 1.

To determine the mechanical properties, one large plate was produced in the direction of 0° with eight layers. Four groups of samples were cut and prepared for tensile, compression, and shear tests to determine the mechanical properties under atmospheric conditions and three different aging conditions. The plates were symmetrically laminated and consisted of different orientation types given in Table 2.

The dimensions of the impact specimens were 120 mm × 170 mm, and the approximate thickness was 2.5 mm. The side edges of the specimens prepared by cutting were painted with epoxy paint before being exposed to aging.

### 2.2. Accelerated Aging

**Figure 2:**
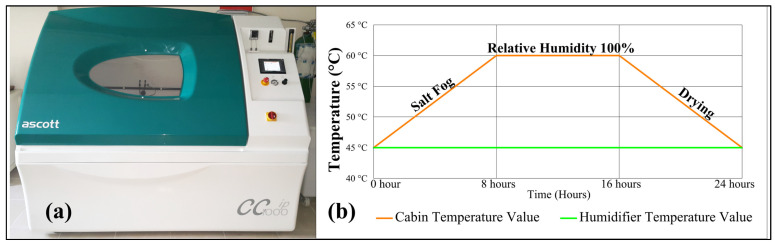
(**a**) Aging cabin, (**b**) One-day accelerated aging process in the cabin.

In this study, the humidity and temperature effects to which composite marine vehicles are exposed were simulated using the ASCOTT CC1000ip (Tamworth, Staffordshire, UK) aging-type device according to ASTM B117 [27] and ISO 9227 [28] standards. The salt fog and cyclic corrosion test cabin used an accelerated aging program to accelerate the aging effect. The carbon/epoxy samples were subjected to the aging effect in three different groups for 2, 4, and 6 weeks. The cabin and one-day program are given in Figure 2, which, as seen, has a 24 h aging cycle that occurs and consists of 3 sections of 8 h. During the first eight hours, the cabin temperature starts from 45 °C and is under the effect of salt fog until it reaches 60 °C. In the second 8 h section, the temperature is constant and under 100% humidity. In the last 8 h, the cabin temperature has dropped to 45 °C, and the samples are in the drying period. The test device contained a 3.5% NaCl saline solution to simulate seawater. The accelerated aging scenario described above has been used by various researchers in their studies in the literature [29,30,31].

The temperature oscillation between 45 °C and 60 °C is a critical parameter in the cyclic corrosion testing protocols according to ASTM B117 and ISO 9227, designed to simulate the realistic diurnal thermal variations and solar heating experienced by marine structures in service. This cyclic thermal loading serves multiple acceleration mechanisms: primarily, it induces repetitive thermal stresses due to the mismatch in the coefficients of thermal expansion between the carbon fibers and the epoxy matrix, thereby facilitating micro-crack initiation at the interface. Additionally, the elevated temperature phase enhances the diffusion rate of moisture and chloride ions into the composite, while the alternating wet and dry cycles promote the crystallization of salt within micro-voids, exerting expansive physical forces that exacerbate matrix degradation and accelerate the overall aging process.

### 2.3. Low Velocity Impact Tests

**Figure 3:**
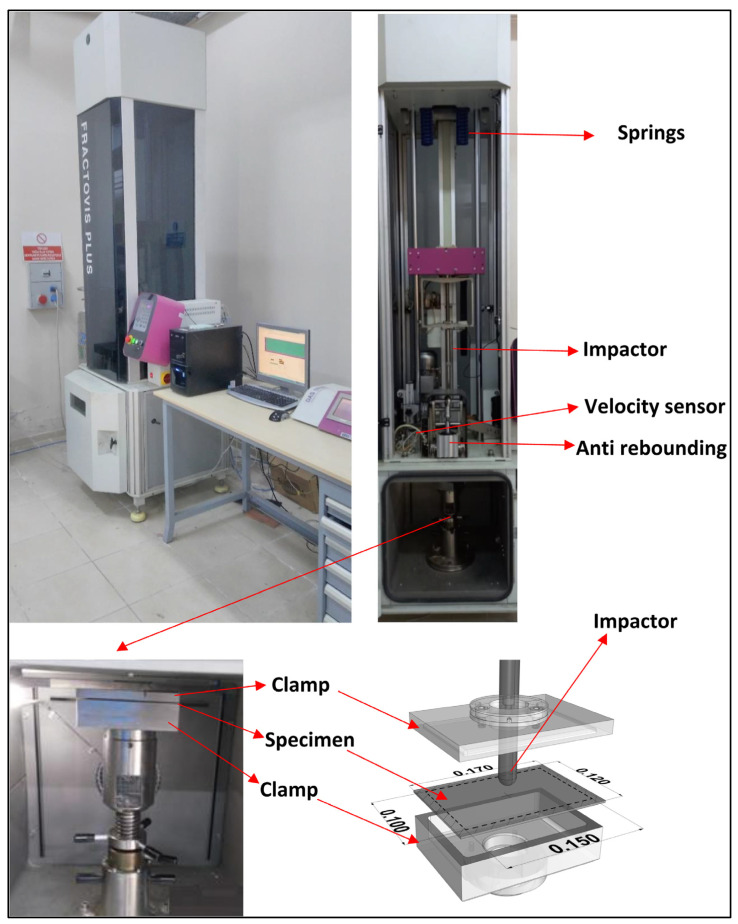
Low velocity impact test setup and samples’ position.

Composite plates were drop-weight impact tested with a CEAST 9350 Fractovis Plus (Turin, Italy) device and a specially designed apparatus for this purpose. The 5.02 kg impactor is made of 0.020 m diameter steel in hemispherical form. Tests were performed at a 30 J impact energy with a velocity of 3.45 m∙s^−1^ for each type of composite plate in four aging conditions (Figure 3). According to the ASTM-D7136/7136M-25 standard [32], rectangular plate specimens are specified for drop-weight impact testing of fiber-reinforced polymer matrix composites.

### 2.4. Compression After Impact (CAI) Tests

**Figure 4:**
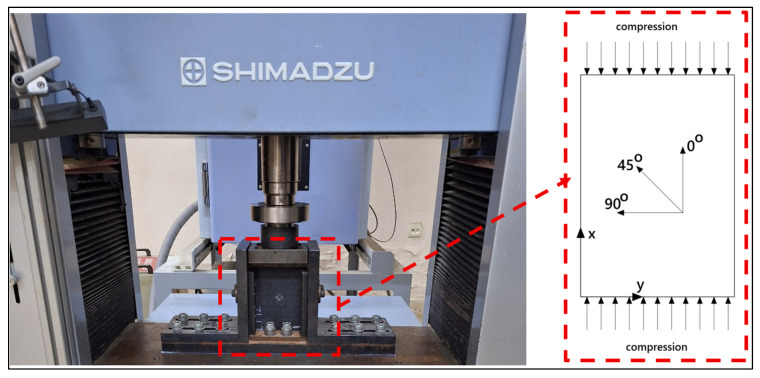
CAI test setup and the specimen view.

In order to determine the effect of damage occurring after low-velocity impact on the strength of the material, the change in the material’s compressive strength was measured using the CAI test apparatus on a Shimadzu AG-X test device (Kyoto, Japan). The apparatus on the test plates was designed to ensure equal distribution of the load and to prevent buckling due to the applied loads and fixed the samples at their four corners (bottom, top, and sides) and the specimen view, as shown in Figure 4. The compression speed of the device was selected as 1 mm/min. The test device can measure and record the applied force, advancement amount, and strain data. Six different laminated plates [0°/90°/45°/−45°]s, [90°/45°/−45°/0°]s, [0°/90°/0°/90°]s, [90°/0°/90°/0°]s, [−45°/45°/−45°/45°]s, and [45°/−45°/45°/−45°]s were tested according to the ASTM.D7137/ D7137M−23 standard [12].

The highest compressive strength (MPa) that the sample can withstand after impact:

(1) $$\sigma_{c}=\frac{F_{m}}{b\;x\;t}$$

where *Fm*, *b*, and *t* are the maximum force before fracture, width of sample (mm), and sample thickness (mm), respectively.

### 2.5. Imaging of Damages

**Figure 5:**
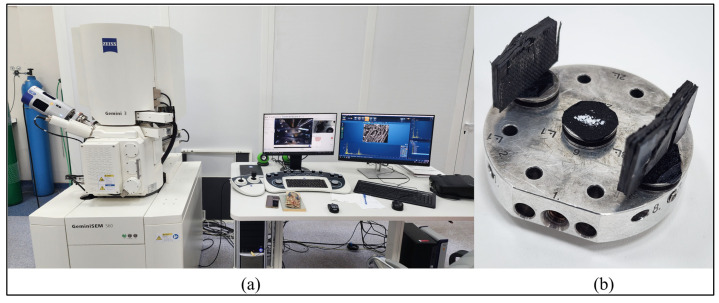
(**a**) The Zeiss GeminiSEM 560 instrument (**b**) Apparatus used for SEM analysis.

To observe the effects occurring before and after aging in conjunction with impact damage, samples of Plate 1’s unaged and six-week-aged conditions were examined using Scanning Electron Microscopy (SEM) and Energy-Dispersive X-ray Spectroscopy (EDS) techniques. To better observe the effect of the impact load, the plates were sectioned at their exact center—where compressive stress was applied—and the impact-damaged region was extracted for analysis. The samples were mounted on the SEM stage in a manner that allows imaging from the cross-sectional surface, and the SEM instrument used was the Zeiss GeminiSEM 560 model (Jena, Germany), illustrated in Figure 5. Images were captured at various magnifications (150×, 500×, 1000×, 1500×, and 2000×) from different layers and fibers of each specimen.

## 3. Results and Discussion

### 3.1. CAI Tests’ Results

**Figure 6:**
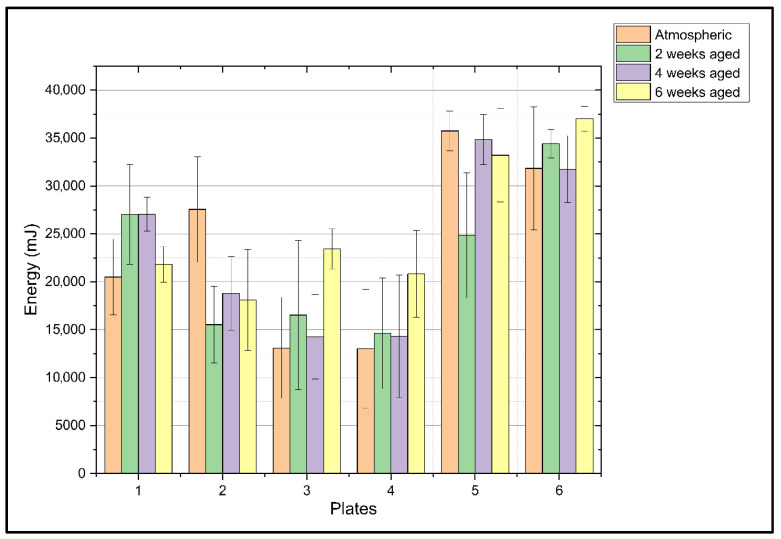
CAI test results.

Figure 6 presents a comparison of the energy dissipated by the manufactured plates under atmospheric and aged conditions, clearly showing significant differences depending on the layering type.

The following conclusions can be easily drawn from this figure:

- Plates 5 and 6, with angle-ply layers, significantly dissipated the most energy, each exceeding 30,000 mJ.
- Plates 3 and 4, with cross-ply layers, exhibited the lowest energy dissipation, approximately 13,000 mJ.
- Thus, plates 1 and 2, with quasi-isotropic layers, achieved an average energy dissipation performance between those of the other two types of plates.

The effects of aging on these values, as well as the energy dissipation values, also vary significantly depending on the layering arrangement. As follows:

- Aging has a positive effect on cross-ply layered plates, increasing their energy dissipation performance. While these plates had the lowest performance of all samples under atmospheric conditions, around 13,000 mJ, their energy dissipation capacity increased with aging by 77% for plate 3 and 100% for plate 4.
- The effect of aging on energy dissipation performance is more complex for plate 1, which is quasi-isotropic, and for plates with angle-ply layering. While plate 1 exhibited similarly high performance (26,000–27,000 mJ) under atmospheric, second-week, and fourth-week aging conditions, these performance values decreased by approximately 20% after 6 weeks of aging. Angle-ply layered plate 5 demonstrated the highest performance under atmospheric conditions, decreasing after two weeks of aging, and aging for 4 and 6 weeks was observed to have a positive effect. Angle-ply array plate 6 demonstrated its highest performance after 6 weeks of aging, corresponding to the highest energy dissipation of all samples at 37,000 mJ.

In cross-ply layered plates, while energy dissipation performance is initially low, this performance increases with aging. This mechanism can be explained as follows: Initially, the layers, especially those along the 0° direction, are quite brittle and undergo brittle fiber fracture, rapidly breaking with low energy dissipation. However, as the matrix ages, particularly when exposed to moisture, it plasticizes, becoming less brittle and more ductile, and the fiber-matrix interface weakens. In fact, this weakening, generally considered negative, also helps prevent brittle fibers from breaking at once. Instead, the failure mode transitions to delamination and fiber pull-out, which have higher energy absorption capacity. As the plate’s strength decreases, its toughness increases, a relationship that can be called the strength-toughness paradox. This can be explained by the effect of crack propagation on energy dissipation in composite materials, where high strength depends on the strength of the fiber-matrix bond. In other words, in a strong bond, in the event of any crack, fiber fracture occurs instead of fiber pull-out, which leads to brittle fracture of the material without allowing energy to be dissipated. Thus, it has a toughness-reducing effect. However, in the materials studied, since the energy is dissipated by debonding, fiber pull-out, and matrix deformation initiated by the crack after impact, toughness decreased as strength increased.

It is reasonable to predict the likely reason why angle-ply layered plates have the highest energy dissipation performance by examining their damage mechanisms. These types of arrays are very effective at dissipating energy through delamination and matrix cracking. As is well known, these failure modes absorb much more energy than fiber fracture. In other words, these plates dissipate energy layer by layer rather than through fracture, resulting in high toughness.

The damage mechanism in quasi-isotropic layered plates is quite complex. For example, plate 1 has a high energy dissipating performance in atmospheric environments, maintaining this performance until the end of the fourth week, but its performance declines after the sixth week. In this plate, the outer layer is oriented at 0°, dissipating and distributing the load and providing high bending rigidity and impact resistance. This plate, notably balanced, has a 90° outer layer, which is matrix-dominant, meaning its mechanical properties depend on the matrix rather than the fibers. These types of layers are highly sensitive to moisture and other environmental influences. During aging, the first microcracks form here, reducing energy absorption performance.

The high standard deviations observed in some results in Figure 6 can be attributed to several factors inherent to composite materials and the complexities of the environmental aging process. First, the stochastic nature of low-velocity impact damage—which involves complex mechanisms such as matrix cracking, fiber breakage, and delamination—leads to inherent variability in the residual compressive strength between individual specimens. Second, the hydrothermal aging process is often non-homogeneous, as moisture molecules penetrate through micro-voids, cracks, and fiber-matrix interfaces at varying rates across different samples. This variability is especially pronounced in matrix-dominant configurations, such as those with 90° outer layers, which are highly sensitive to environmental wear and prone to localized micro-cracking. Furthermore, the transition from brittle fiber fracture to more energy-dissipating modes like fiber pull-out and delamination during matrix plasticization may progress unevenly among aged samples. Finally, localized surface degradation, including irregular salt accumulation and resin peeling as revealed by SEM and EDS analyses, creates inconsistent stress concentrations that further contribute to the scattering of experimental data.

### 3.2. Damage Mechanism Analysis with Images

**Figure 7:**
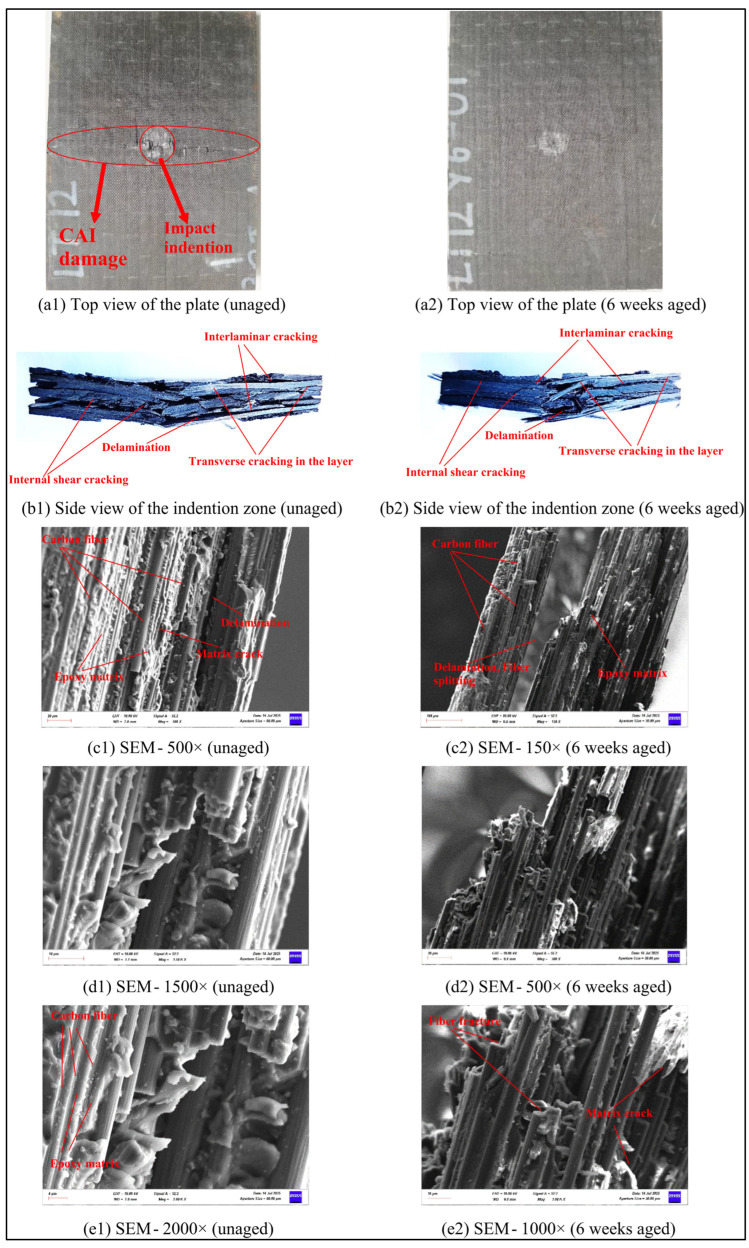
Digital photos and Scanning Electron Microscopy (SEM) images of unaged and 6-week-aged CFRP plates.

From the SEM images obtained and presented in Figure 7, clues can be found regarding the accuracy of the estimates made in the previous section regarding the damage suffered by the samples after CAI tests.

Interlaminar cracking, delamination, internal shear cracking, and transverse cracking in the layer were observed in both unaged and six-week-aged specimens in Figure 7(b1,b2).

After six weeks of aging, it was observed that the degraded epoxy resins on the specimen’s surface had bonded together, forming long matrix cracks as shown in Figure 7(c2,e2).

With electron microscope scanning (SEM) at 150×–500× zoom, carbon fiber and epoxy matrices are visible in Figure 7(c1,c2). Matrix cracks, delamination failure between the two layers of carbon fibers, and fiber splitting are visible at 500×, 1000×, 1500×, and 2000× zooms. Fiber breakage, matrix cracks, and fiber-matrix debonding failure are more clearly visible in Figure 7(d1,d2,e1,e2). It can be said that all these damages reduce the CAI strength of the CFRP material.

Moreover, it was observed that the cracks merged on the front side of the sample and formed a circumferential fracture, consistent with the literature [33].

It was shown that salt particles progressively accumulated on the surface of the composites in the simulated seawater environment. Moreover, certain resin blocks were seen to peel away, exposing fibers, and the carbon fiber had debonded from the resin.

**Figure 8:**
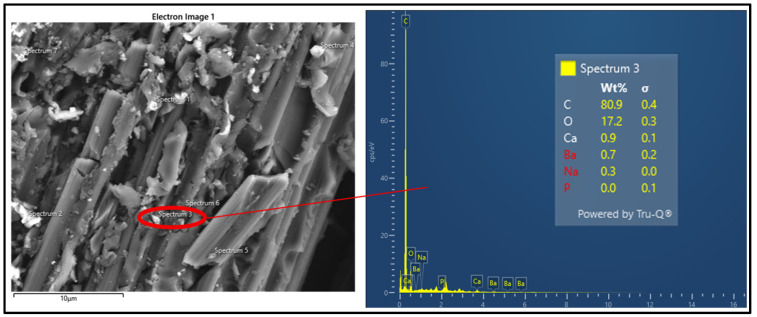
Energy-Dispersive X-ray Spectroscopy (EDS) analysis.

Subsequently, an EDS analysis was performed on the aged sample to detect the presence of corrosion. As expected, the data obtained from eight different spectra revealed a dominant presence of carbon and oxygen, along with detectable amounts of calcium and sodium. The EDS analysis results are shown in Figure 8. The EDS analysis shows that Na and Ca compounds accumulated on the surface of the specimen subjected to 6 weeks of artificial aging shown in Figure 8.

The aging process reveals the degradation process of artificial seawater on the specimen. As this time increases, it penetrates the center of the specimens, causing deterioration of mechanical properties. Some researchers have indicated that the dominant failure mode in CAI tests is buckling-induced interlaminar delamination [34]. This study also shows that fiber breakage and matrix cracking are important.

## 4. Conclusions

This study comprehensively evaluated the CAI performance of CREC under the combined effects of lamination sequences and hydrothermal aging. The experimental results demonstrated that lamination orientation plays a decisive role in the damage tolerance of marine composites.

A significant finding of the research is the complex impact of marine aging on the material’s mechanical behavior. While hydrothermal exposure led to matrix plasticization and a subsequent reduction in peak impact forces, it also triggered a shift in failure mechanisms from brittle fiber fracture to more energy-absorbent modes such as fiber pull-out and extensive delamination. Consequently, in certain sequences, aging surprisingly resulted in a slight increase in post-impact strength by mitigating stress concentrations through localized softening. However, high standard deviations in aged specimens, particularly in matrix-dominant layouts, highlight the non-homogeneous nature of environmental degradation. These findings provide critical design data for the structural health and safety of high-speed marine vessels, emphasizing that both stacking sequences and long-term environmental exposure must be integrated into the preliminary design phase to ensure reliability under severe operational conditions.

Angle-ply layered plates generally exhibit the highest energy dissipation performance.

It has become clear that the effects of aging cannot be unidirectionally determined; in other words, aging cannot be generally defined as “good” or “bad.” While the performance of some quasi-isotropic materials decreases with age, the performance of cross-ply layered plates increases. This can be explained by internal structural changes, such as the post-coring effect or the effects of aging at the interface.

In angle-ply layered plates consisting of only ±45° layers, energy is perfectly dissipated through delamination. In cross-ply layered plates, the layers are normally brittle, but the ductility resulting from aging enhances energy absorption performance. In quasi-isotropic plates, performance varies depending on the outer layer. If the outer layer is 90°, performance decreases, while at 0°, the plate is much more stable and durable.

One of the ways to increase the impact resistance is hybridization, where two or more types of reinforcements or matrices, or both, are used, one with a high modulus and the other with a low modulus. Also, using nanomaterials such as carbon nanotubes or graphene as an additive matrix material or using already toughened resins will increase the impact resistance of the matrix material.

## Figures and Tables

**Table 1:** Physical and mechanical properties of components of carbon/epoxy composite [26].

Property	Fiber	Matrix
	DowAksa Carbon Fiber 24K-A42-1600	FBRMAKRES 11564 Epoxy Resin
Tensile Strength	4200 MPa	70–75 MPa
Tensile Modulus	240 GPa	2850–3000 MPa
Density	1.80 g∙cm^−3^	1.1–1.2 g∙cm^−3^

**Table 2:** Laminates with their sequence, definition, and characterization.

Laminate	Sequences	Definition	Characterization
**1**	[0°/90°/45°/−45°]s	Quasi-isotropic	Jack-of-all-trades laminates offer balanced rigidity and strength properties in all directions.
**2**	[90°/45°/−45°/0°]s		
**3**	[2(0°/90°)]s	Cross-ply	Anisotropy is high. Although they have sufficient strength in the axial direction (0°), they are weaker in the 90° direction.
**4**	[2(90°/0°)]s		
**5**	[2(−45°/45°)]s	Angle-ply	In the axial direction (0°), they are not very stiff under tensile loads. Their modulus of elasticity is low, and they are resistant to shear loads.
**6**	[2(45°/−45°)]s		

## Data Availability

The original contributions presented in this study are included in the article. Further inquiries can be directed to the corresponding author.
